# Supplementary material for: HIVprotI: an integrated web based platform for prediction and design of HIV proteins inhibitors
Source: J Cheminform. 2018 Mar 9;10:12. doi: 10.1186/s13321-018-0266-y (PMC5845081; doi:10.1186/s13321-018-0266-y)
Supplement: Supplementary file 2 — Additional file 2. Source code of HIVProtI web server. [file 13321_2018_266_MOESM2_ESM.zip › HIVprotI_Source-code/hivprotI/weka-3-6-6/doc/weka/gui/graphvisualizer/GraphEdge.html]

GraphEdge


---


|  |  |  |
| --- | --- | --- |
| **Overview** \| **Package** \| **Class** \| **Tree** \| **Deprecated** \| **Index** \| **Help** \| **Weka's home** | |  |
| **PREV CLASS**   **NEXT CLASS** | **FRAMES**    **NO FRAMES**     **All Classes** |  |
| SUMMARY: NESTED \| FIELD \| CONSTR \| METHOD | DETAIL: FIELD \| CONSTR \| METHOD |  |


---


## weka.gui.graphvisualizer Class GraphEdge

```
java.lang.Object
  weka.gui.graphvisualizer.GraphEdge
```

---

``` public class GraphEdge extends java.lang.Object ```

This class represents an edge in the graph

**Version:**
:   $Revision: 1.4 $ - 23 Apr 2003 - Initial version (Ashraf M. Kibriya)

**Author:**
:   Ashraf M. Kibriya (amk14@cs.waikato.ac.nz)

---

**Constructor Summary**

`GraphEdge(int s,
int d,
int t)`

`GraphEdge(int s,
int d,
int t,
java.lang.String sLbl,
java.lang.String dLbl)`


| **Method Summary** | |
| --- | --- |
| `boolean` | `equals(java.lang.Object e)` |
| `java.lang.String` | `toString()` |

**Methods inherited from class java.lang.Object**

`getClass, hashCode, notify, notifyAll, wait, wait, wait`

**Constructor Detail**

### GraphEdge

```
public GraphEdge(int s,
                 int d,
                 int t)
```

---


### GraphEdge

```
public GraphEdge(int s,
                 int d,
                 int t,
                 java.lang.String sLbl,
                 java.lang.String dLbl)
```


**Method Detail**

### toString

```
public java.lang.String toString()
```

:   **Overrides:**: `toString` in class `java.lang.Object`

---


### equals

```
public boolean equals(java.lang.Object e)
```

:   **Overrides:**: `equals` in class `java.lang.Object`


---


|  |  |  |
| --- | --- | --- |
| **Overview** \| **Package** \| **Class** \| **Tree** \| **Deprecated** \| **Index** \| **Help** \| **Weka's home** | |  |
| **PREV CLASS**   **NEXT CLASS** | **FRAMES**    **NO FRAMES**     **All Classes** |  |
| SUMMARY: NESTED \| FIELD \| CONSTR \| METHOD | DETAIL: FIELD \| CONSTR \| METHOD |  |


---
